# Hexaferrocenium tri[hexa(isothiocyanato)iron(III)] trihydroxonium complex as a new DNA intercalator for electrochemical DNA biosensor

**DOI:** 10.1038/s41598-021-86939-z

**Published:** 2021-04-12

**Authors:** Eda Yuhana Ariffin, Emma Izzati Zakariah, Farah Ruslin, Muhammad Kassim, Bohari M. Yamin, Lee Yook Heng, Siti Aishah Hasbullah

**Affiliations:** Department of Chemical Sciences, Faculty of Science and Technology, Universiti Kebangsaan Malaysia (UKM), 43600 Bangi, Selangor Darul Ehsan Malaysia

**Keywords:** Chemistry, Materials science

## Abstract

Ferrocene or ferrocenium has been widely studied in the field of organometallic complexes because of its stable thermodynamic, kinetic and redox properties. Novel hexaferrocenium tri[hexa(isothiocyanato)iron(III)]trihydroxonium (HexaFc) complex was the product from the reaction of ferrocene, maleic acid and ammonium thiocyanate and was confirmed by elemental analysis CHNS, FTIR and single crystal X-ray crystallography. In this study, HexaFc was used for the first time as an electroactive indicator for porcine DNA biosensor. The UV–Vis DNA titrations with this compound showed hypochromism and redshift at 250 nm with increasing DNA concentrations. The binding constant (K_b_) for HexaFc complex towards CT-DNA (calf-thymus DNA) was 3.1 × 10^4^ M^−1^, indicated intercalator behaviour of the complex. To test the usefulness of this complex for DNA biosensor application, a porcine DNA biosensor was constructed. The recognition probes were covalently immobilised onto silica nanospheres (SiNSs) via glutaraldehyde linker on a screen-printed electrode (SPE). After intercalation with the HexaFc complex, the response of the biosensor to the complementary porcine DNA was measured using differential pulse voltammetry. The DNA biosensor demonstrated a linear response range to the complementary porcine DNA from 1 × 10^−6^ to 1 × 10^−3^ µM (R^2^ = 0.9642) with a limit detection of 4.83 × 10^−8^ µM and the response was stable up to 23 days of storage at 4 °C with 86% of its initial response. The results indicated that HexaFc complex is a feasible indicator for the DNA hybridisation without the use of a chemical label for the detection of porcine DNA.

## Introduction

Ferrocene is a stable metal complex and consists an Iron(II) atom sandwiched between two cyclopentadienyl ligands^1^. Ferrocene has been commonly studied due to its properties and applications in research comprising organic synthesis, catalyst and materials science^2^. Ferrocene when loses an electron, ferrocenium species are formed resulting in the oxidation state to increase from 2^+^ to 3^+^. Ferrocenium is a free radical species of good stability as it has an unpaired electron in one of the nonbonding orbitals. Hence, because of all of these features, ferrocene and ferrocenium complexes are a good agent that is often used in biomedical fields^3^.

One of the usefulness of ferrocenium complexes and derivatives in biomedical fields is as an indicator for DNA detection. This characteristic is attributed to the electrochemical properties of the remarkable and straightforward redox-active unit of the ferrocene^4^. In addition, it has attracted a lot of attention of many researchers because it is an inexpensive but stable compound for a DNA detection system. The differential of the indicator’s concentration before and after DNA hybridisation is often related to the voltammetry peak current. Liu et al.^4^ had employed the interaction between DNA and ferrocenium complex interaction based on the Langmuir–Blodgett film to develop a DNA electrode. Ferrocenium complex was used as an electroactive indicator to detect dengue DNA^5^. Ju et al.^6^ have reported a DNA hybridisation indicator using ferrocene derivatives to label the yeast DNA chain. Ferrocenylnaphthalenediimide was used as DNA hybridisation intercalator to detect Sus scrofa mtDNA in food adulteration^7^. Sato and Takenaka^8^ studied electrochemical DNA detection using ferrocenylnaphthalenediimide. In most of the reported work involving ferrocene/Fe(III) compounds of DNA hybridisation indicator, they were used as labels that needed to be chemically attached to the DNA probes to detect a hybridisation event^9,10^.

Pork is typically involved in meat adulteration for example it is added to beef because pork is cheaper than beef. Pork is also frequently added into beef and chicken meatballs as additive^11^. It is difficult and impossible in differentiating between beef and pork by visual inspection^12^. Fish breeders also use boiled pork intestines and internal organs to feed their fish^13^. Some religious laws strictly-forbidden the existence of pork and its by-products in foods^14^. It is difficult for the consumer to identify the product containing of pork and its by-product. Thus, the assessment of the possible appearance of pork meat in food is a major consumer’s demand for protection against falsely labelled food. A porcine DNA biosensor is one way to overcome the detection of pork in food as DNA biosensors are easily manageable, transportable, rapid detection, and a verification method for pork detection.

In this work, a novel compound, hexaferrocenium tri[hexa(isothiocyanato)iron(III)]trihydroxonium (HexaFc) complex was utilised as intercalator in the development of a DNA electrochemical biosensor. The hexaferrocenium cation and hexa(isothiocyanato)iron(III) of HexaFc complex are expected to interact with the DNA via intercalation mode during the hybridisation event and allowed a direct detection of DNA hybridisation without the need of any further DNA label attachment procedure. The uniqueness of this complex for DNA recognition permitted us to develop a porcine DNA biosensor. The HexaFc complex was covalently immobilised onto silica nanospheres (SiNSs) via glutaraldehyde linkers. Gold nanoparticles (AuNPs) were used to improve the overall electrochemical response of the DNA biosensor^15^. Differential pulse voltammetry was used to measure the current changes in response to complementary porcine DNA in the presence of HexaFc complex as DNA intercalator.

## Methods

### Apparatus

Field emission scanning electron microscope (FESEM) images were recorded in FESEM model Zeiss SUPRA 55VP with Energy-dispersive X-ray Analysis (EDX). The crystalline structure was assessed by X-ray diffraction (Cu K_α_ radiation, λ = 0.15416 nm; 0.025° 0.1 s^−1^) (model D8 Advance, brand Bruker AXS Germany). DNA titration were recorded using Varian Cary 50 UV–Vis Spectrophotometer. Differential pulse voltammetry (DPV) measurements were carried out using AUTO-LAB PGSTAT and NOVA software package. A three-electrode system using Ag/AgCl as a reference electrode (Metrohm), platinum electrode as the counter electrode and screen-printed electrode (SPE) as the working electrode. The electrochemical measurements were carried out in 0.05 M potassium phosphate buffer solution (PBS) at pH 7 and were scanned from − 0.4 to 0.4 V with a step potential of 0.01 V.

### Reagents and materials

Synthetic oligonucleotides, calf-thymus DNA (CT-DNA) and the reagents (3-aminopropyl)triethoxysilane (APTES, 99%), glutaraldehyde, DNA, gold nanoparticles (AuNPs, <100 nm diameter), potassium chloride (KCl), ferrocene, maleic acid, ammonium thiocyanate) were supplied by Sigma Aldrich. The dihydrogen phosphate (KH_2_PO_4_) and potassium ferricyanide (K_3_[Fe(CN)_6_]) chemicals were received from Merck, tetraethyl orthosilicate (TEOS, 98%) was obtained from Fluka, ammonia (25%) and ethanol (95%) from Systerm. Besides, dipotassium hydrogen phosphate (K_2_HPO_4_) was supplied by BDH Laboratory Supplies. MilliQ-deionized water was used to prepare all standard buffer and chemical solutions. The DNA oligonucleotide sequences used in the present research:

Porcine DNA probe (100 OD): 5′-CTG ATA GTA GAT TTG TGA TGA CCG TAG [AmC3]

Complementary Porcine DNA: 5′-CTA CGG TCA TCA CAA ATC TAC TAT CAG

Beef taurusa cytb: 5′-CAT AGC AAT TGC CAT AGT CCA CCCTA

Gallus gallus cytb: 5′-CGC AGG TAT TAC TAT CAT CCA CC

### Synthesis and DNA binding of hexaferrocenium complex

An ethanolic solution of ferrocene (1.864 g; 0.01 mol) was added into 50 mL solution mixture of maleic acid (1.260 g; 0.01 mol) and ammonium thiocyanate (1.5224 g; 0.02 mol). The mixture was heated and stirred for 30 min. The excess ferrocene was filtered out, and the mixture was left to crystallise at room temperature. Bluish-black shiny needle-like crystal was formed after one day. Yield: 29%. melting point: 171–172 °C. Analysis: Calcd (%): C 39.17, H 3.00, N 10.54, S 24.11. Found (%): C 40.89, H 2.98, N 8.19, S 23.02 IR (cm^−1^): 3099 ν(C–H), 1413 ν(C–C), 1007 δ(C–C), 852 δ(C–C), 2050 ν(NCS).

5 mM of Tris-HCl buffer at pH 7.1 was used to prepare CT-DNA stock solution. UV–Vis spectra were recorded in 1 cm path length of quartz cuvettes using a Shimadzu UV-1800 spectrophotometer. The ratio of the absorbance of the CT-DNA solution at 260 nm and 280 nm was 1.87, which was more than 1.8, indicating that CT-DNA was not contaminated by proteins. A molar absorption coefficient value of 6600 M^−1^ cm^−1^ was applied to calculate the concentration of CT-DNA from its absorption intensity at 260 nm^14^.

### Synthesis of aminated SiNSs

Aminated silica nanospheres (SiNSs) were prepared as per-described by Sani et al.^16^. TEOS (2 mL) and ethanol (20 mL) was added into mixture of deionized water (2 mL), ammonium solution (5 mL) and ethanol (20 mL), and sonicated for 40 min at 55 °C. Next, the mixture was treated with 2 mL of APTES (99%) and leave 24 h stirring. The aminated SiNSs solution was washed sequentially with ethanol and deionized water by centrifugation of 4000 rpm for 20 min each. The aminated SiNSs slurry were collected and air-dried overnight.

### Fabrication and characterisation of DNA biosensor

**Figure 1:**
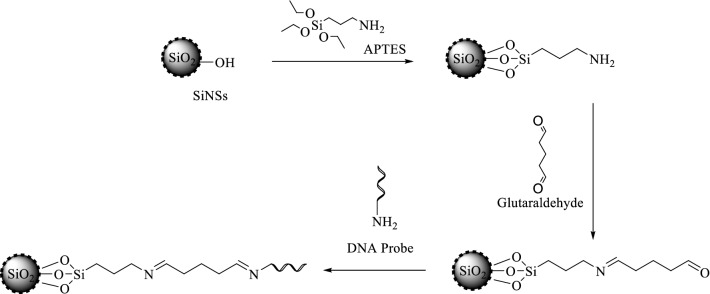
Proposed chemical reaction for the fabrication of porcine DNA probe.

**Figure 2:**
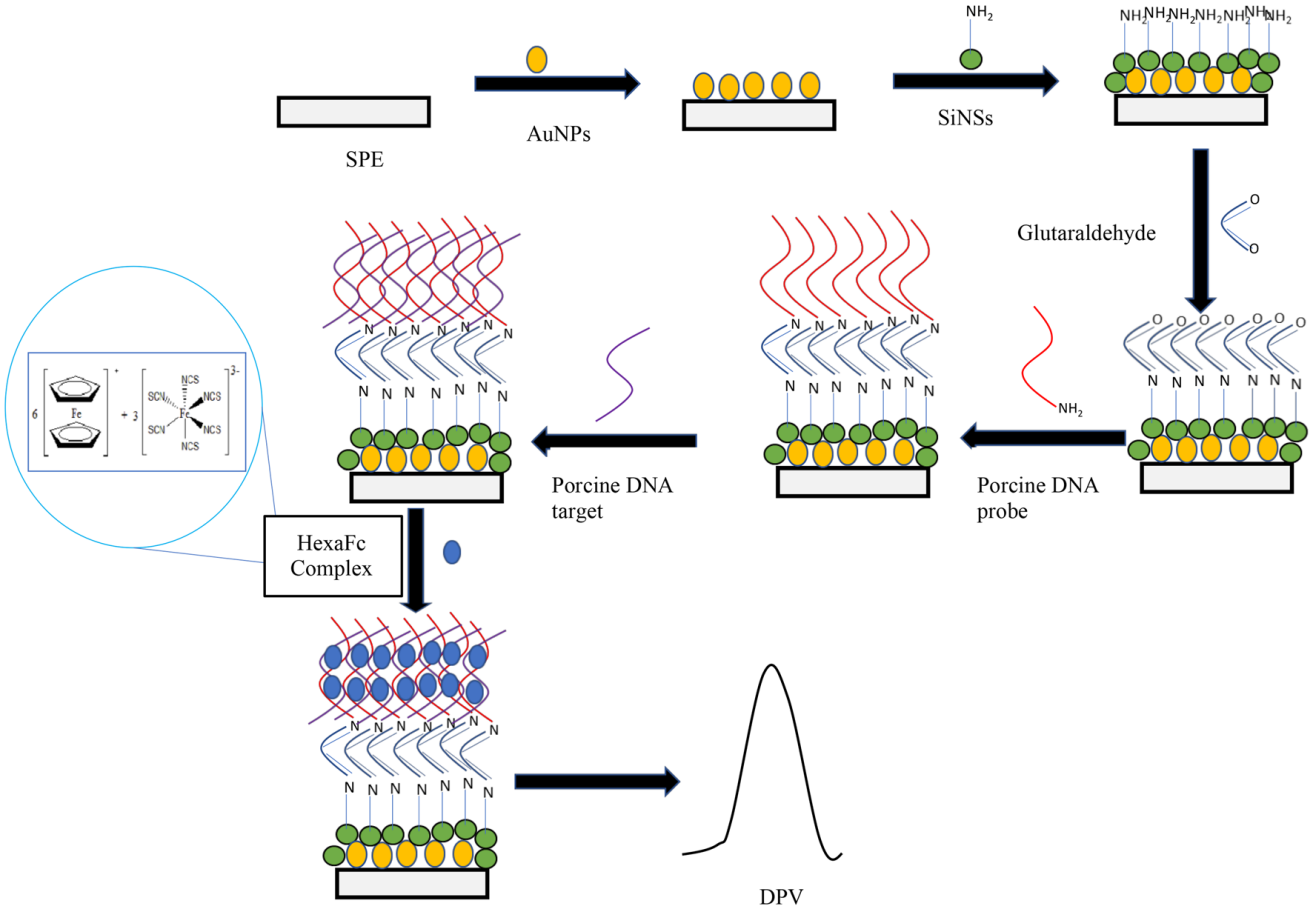
Fabrication of porcine DNA biosensor using HexaFc as DNA hybridisation marker.

The SPE electrode was first deposited with 10 µL of colloidal AuNPs (0.005 mg/µL) and dried at room temperature. Then, 5 µL of 2 mg SiNSs in 300 µL of ethanol was deposited onto the AuNPs-modified SPE, air-dried and immersed in 10% glutaraldehyde for 1 hour. The fabricated probe was immersed in 300 µL of porcine DNA probe solution (1 µM) for 24 hours. The biosensor was then rinsed with 0.05 M potassium phosphate buffer, pH 7 and hybridised in 300 µL of complementary porcine DNA solution for 1 h. The DNA was rinsed again with 0.05 M potassium phosphate buffer, pH 7 followed by dipping it in 2 × 10^−5^ M HexaFc solution for 1 h and the response of the DNA biosensor was scanned using DPV. The proposed chemical reaction is shown in Fig. 1. The fabrication of porcine DNA biosensor using HexaFc as a DNA hybridisation marker and shown in Fig. 2. This fabrication is quite similar to Mishra et al.^17^. The functional group of biosensors was determined by Fourier transform Infared Spectroscopy. The morphology of the SiNSs-DNA was determined by FESEM and FESEM-EDX (energy-dispersive X-ray) elemental mapping with platinum coating by sputtering. Meanwhile, the X-ray diffraction study of the DNA biosensor was performed using powder X-ray diffraction (XRD). The diffraction angle was measured with the X-ray radiation of Cu Kα.

### Performance evaluation of electrochemical porcine DNA biosensor

The scan rate study was carried out to understand the nature of the reaction. Scanning rate studies of modified SPE electrodes were performed in K_3_[Fe(CN)_6_] at 1 mM using cyclic voltammetry (CV) techniques at scan rates of 10–300 mV s^−1^. The deposited layer on the electrode surface was characterised by CV at a scan rate of 100 mV s^−1^ by using 2 × 10^−4^ M HexaFc complex as a redox label. The linear range of the porcine DNA biosensor was obtained by using a series of complementary porcine DNA concentration between 1 × 10^−7^ µM until 1 × 10^−2^ µM with constant HexaFc complex concentration at 2 × 10^−5^ mM. The selectivity of DNA biosensor with complementary porcine DNA and non-complementary DNA was examined at the same concentration. The new porcine DNA biosensor was tested with raw pork samples. Pork DNA from raw meat was extracted according to the Spin-Column Protocol by using a DNeasy kit. The DNA obtained was hybridised with the immobilised DNA sequence of porcine on the biosensor in the presence of the new DNA redox indicator. The signal received was compared to the unhybridised response of the biosensor.

## Results and discussion

### Infra-red spectroscopy and crystal structure

**Figure 3:**
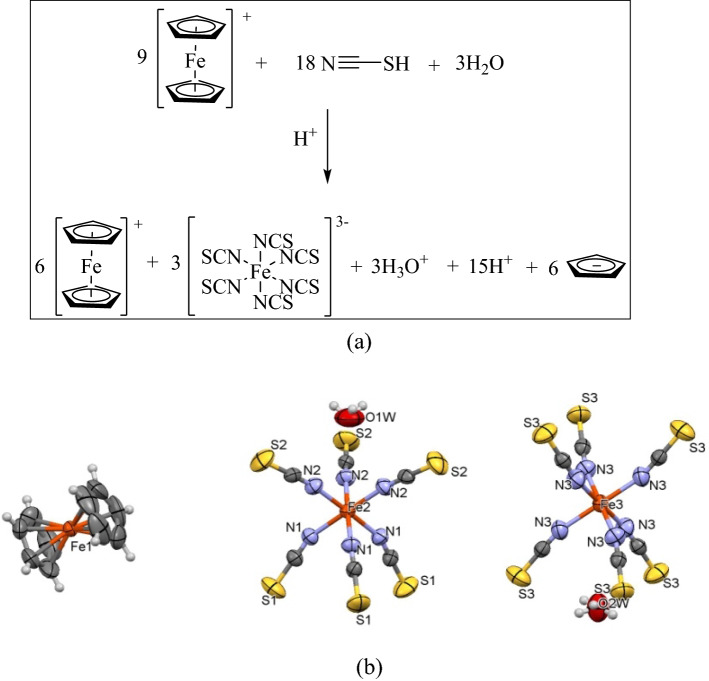
(**a**) Structure of HexaFc, (**b**) HexaFc crystal structure with 30% ellipsoid probability at c-axis.

Five significant peaks of ferrocenium compound at 2050.63, 3099.75, 1413, 1007 and 852 cm^−1^ were observed. The presence of a sharp stretching peak at frequency 2050.63 cm^−1^ is owing to the N=C=S. Meanwhile the peak at 3099.75 cm^−1^ is a characteristic for CH stretching of the cyclopentadienyl rings for ferrocenium group. The peak at 1413 cm^−1^ corresponds to the antisymmetrical C–C stretching while the peak at 852.17 cm^−1^ represents a CH out of plane mode for ferrocenium. The presence of all these significant peaks indicates that this ferrocenium compound has successfully formed^18^ (Fig. 3a).The compound crystallized in trigonal crystal system with space group P-3, a = b = 18.1606(12) Å, c = 8.9808(6) Å, α = β = 90°, γ = 120°, Z = 1 and V = 2565.1(4). CCDC number for this compound is 1914296. From X-ray crystallography investigation, the titled compound consisted of six ferrocenium moiety, three hexa(isothiocyanato)iron(III) complexes and three hydronium group (Fig. 3). The positive charge was stabilised by the presence of three hexa(isothiocyanato)iron(III) complexes and three hydronium group. All hexa(thiocyanato)iron(III) complexes in the compound adopt octahedral structure (Fig. 3b).

### DNA binding of hexaferrocenium complex

**Figure 4:**
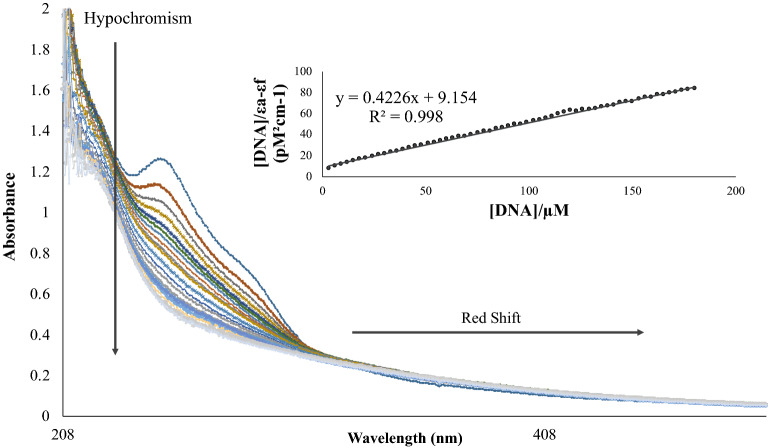
Absorption spectral changes of HexaFc complex (30 μM) in Tris-HCl buffer (pH 7.1) in the absence and presence of increasing concentrations of CT-DNA. Inset: fitting of the absorbance data that were used to obtain the binding constant at 250 nm.

The binding mode and the binding affinity between HexaFc complex and CT-DNA were performed using UV–Vis spectroscopic titrations. The UV–Vis absorption spectra of the title compound show one intense bands at 250 nm (Fig. 4). The absorption band at 250 nm is assigned to the π–π* transition, which attributes to the localisation of molecular orbitals on the C=C group of hexaferrocenium cation. The absorption band at 280 nm is referring to the n–π* transition of C=N of hexa(isothiocyanato)iron(III) anion. Absorption measurements were carried out by using constant HexaFc complex concentration (3 × 10^−5^ M) while increasing the concentration of DNA until no changes could be seen on the UV–Vis spectrum. The spectrum of the HexaFc complex was recorded after each addition of the DNA.

Changes that could be observed in the spectrum were either hyperchromism (increased in absorption) or hypochromism (decreased in absorption). Hyperchromism occurs due to the secondary damage of the double helix structure of the DNA, causing the DNA to be single-stranded^19,20^. The occurrence of hypochromism is caused by the contraction of DNA in the helical axis. It is also affected by the transformation in DNA conformation^21^. Besides, the change in the wavelength of either the redshift (the absorption to the longer wavelengths) or the blue shift (the absorption change to the shorter wavelength) can also be observed.

The percentage of hypochromicity was calculated following Equation (1):

1 $$\%Hypochromicity=\frac{{\varepsilon }_{free}-{\varepsilon }_{bound}}{{\varepsilon }_{free}}\times 100$$

Based on the UV–Vis DNA titrations, this compound exhibited a change in hypochromism and redshift at 250 nm when DNA concentration increased. The hypochromicity shown was 40%, and the redshift was 2.5 nm and in agreement with the established intercalators^22,23^. According to Wu et al.^24^ and Shahabadi et al.^25^, hypochromism event showed the binding strength of compounds towards DNA through intercalation mode. DNA binding through intercalation causes redshift. This involves strong overlapping interactions between the aromatic ligand chromophores of the metal complex with DNA bases^26,27^. Meanwhile, the effects of hyperchromism or hypochromism can be observed when the occurrence of electrostatic interaction or groove binding followed by a blue shift (hypsochrome effect) or a minor change in the wavelength absorption of the UV–Vis spectrum^28^. The intrinsic binding constant K_b_ of hexaferrocenium salt–DNA was determined according to Eq. (2):

2 $$\frac{[DNA]}{{(\varepsilon }_{A}-{\varepsilon }_{F})}=\frac{[DNA]}{{(\varepsilon }_{B}-{\varepsilon }_{F})}+\frac{1}{{K}_{b}({\varepsilon }_{B}-{\varepsilon }_{F})}$$

where the apparent molar extinction coefficients, Δε_ap_ = |ε_A_ − ε_F_|, ε_A_ = A_observed_/[Complex], Δε = |ε_B_ − ε_F_|. ε_F_ and ε_B_ represent molar extinction coefficients for the free hexaferrocenium salt and the DNA bound hexaferrocenium complex, respectively. To interpret the binding affinity of the compound to DNA, the intrinsic binding constant K_b_ was discovered by recognising the changes of maximum absorption bands centred at around 250 nm region. From the plotted graph of [DNA]/|ε_A_ − ε_F_| versus [DNA], the y-intercept is equal to 1/(|ε_B_ − ε_F_| × K_b_) whereas the slope is equal to 1/|ε_B_ − ε_F_|. K_b_ values can be determined by dividing the slope value by the y-intercept. The binding constant (K_b_) for HexaFc complex towards CT-DNA was 3.1 × 10^4^ M^−1^. Also, this compound exhibited the same approximation value of binding constant with other reported ferrocene derivatives towards CT-DNA^29,30^.

### Characterisation of DNA biosensor

**Figure 5:**
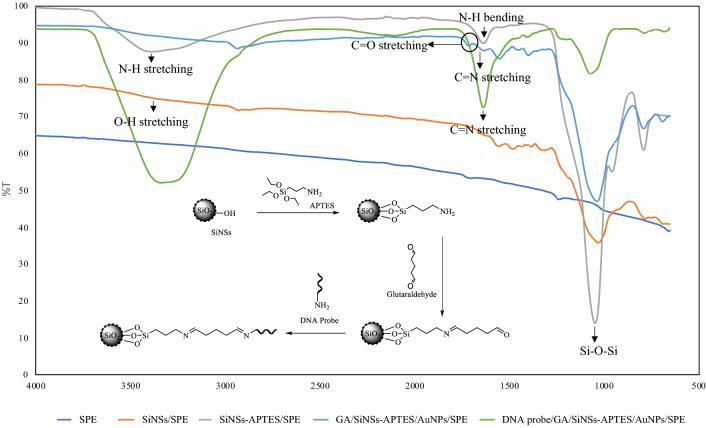
FTIR spectra of DNA biosensor.

We observed the characteristic modification when SiNSs immobilised porcine DNA probe and interacted with complementary DNA molecules. Infrared spectroscopy was used to characterise the features of SiNSs before and after the reaction with APTES and before and after the immobilization of DNA probe. The FTIR spectra for the DNA biosensor shown in Fig. 5. After silica was modified with APTES, a sharp and strong stretching peak at ∼ 1039 cm^−1^ was observed to indicate Si–O–Si stretching frequency. The peak observed at ∼ 1543 cm^−1^ (N–H bending) and ∼ 3400 cm^−1^ (N–H stretching) implied the presence of NH_2_ group. Further react with glutaraldehyde resulted in the formation of a peak at ~ 1630 cm^−1^ (C=N stretching) and ~ 1715 cm^−1^ (C=O stretching) and disappearance of a peak at ∼ 3400 cm^−1^ (N–H stretching) which indicated the formation of imine and presence of aldehyde groups. After the aminated DNA probe was immobilized onto GA/SiNS-APTES/AuNp/SPE, a sharp and clear FTIR adsorption band was formed at ~ 1620 (C=N stretching), which represent for imine group. The less intense peak formation might due to the using of a small quantity of each layer since this spectrum results from the fabrication on the screen-printed electrode.

**Figure 6:**
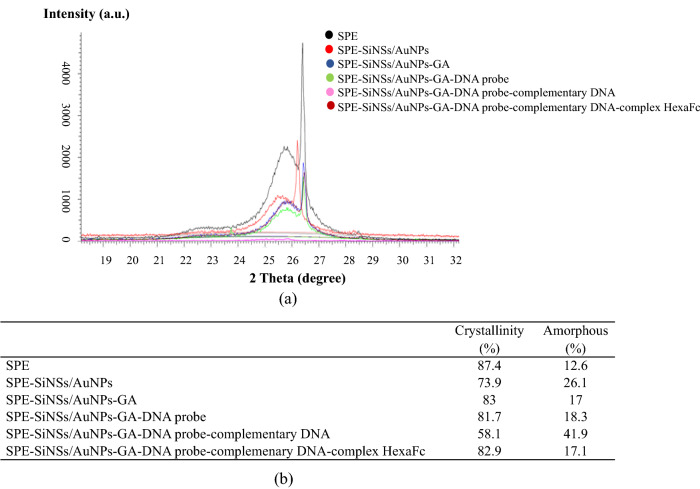
(**a**) XRD diffractogram and (**b**) crystallinity table of layer-by-layer DNA biosensor.

The XRD patterns of layer-by-layer DNA biosensor are shown in Fig. 6. The diffractogram of SPE exhibited a band at 25.6°. The intensity band and the percentage of crystallinity decreased after AuNPs and SiNSs were deposited on SPE. But the intensity band and the percentage of crystallinity were slightly increased after treatment with glutaraldehyde (GA)^31^. The intensity band and the percentage of crystallinity continued to decrease with the addition of the porcine DNA probe and the complementary DNA. After the addition of HexaFc complex, the intensity and the percentage of crystallinity began to increase due to the presence of metal iron from the complex.

**Figure 7:**
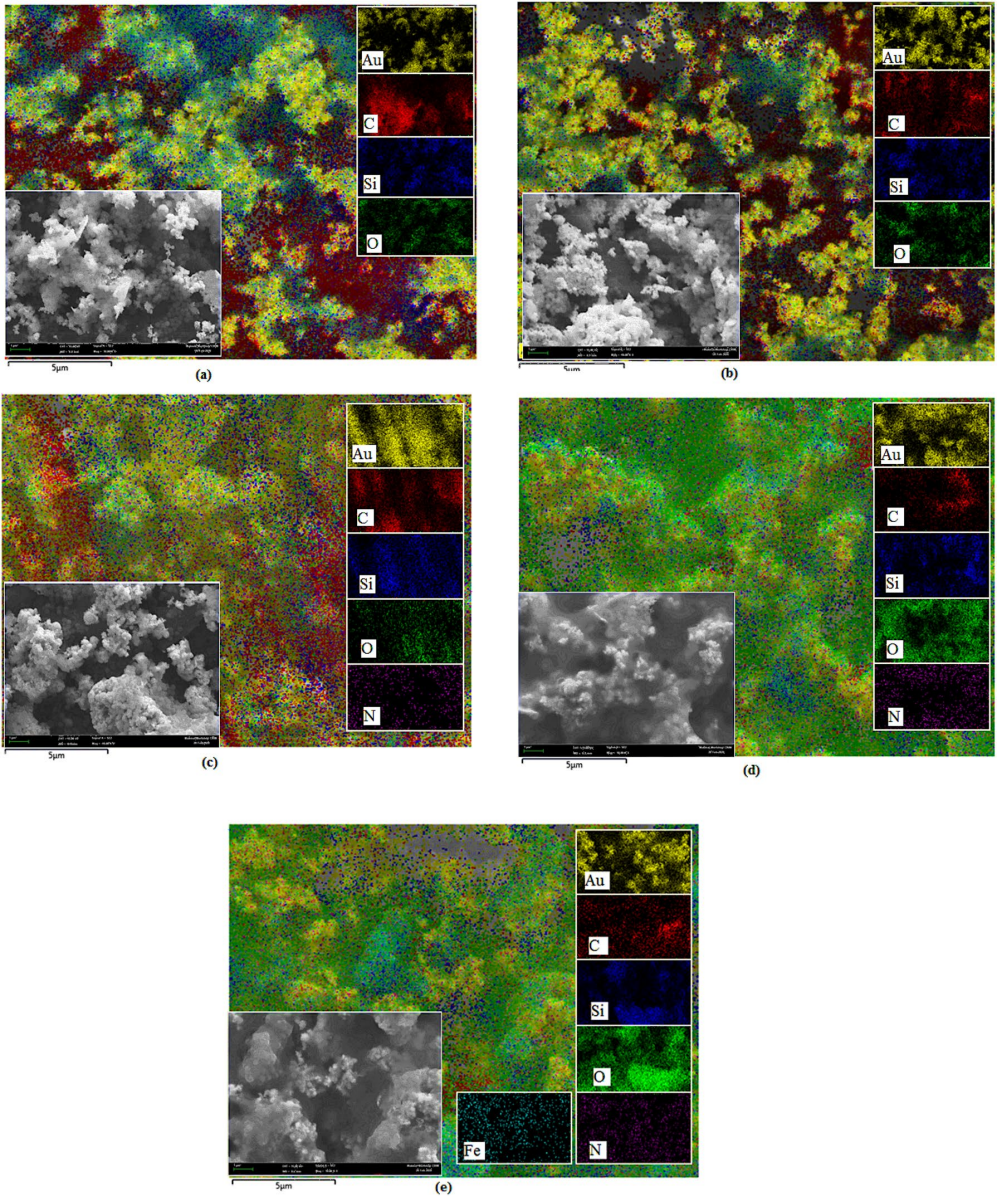
EDX elemental mapping analysis. Inset: FESEM micrograph of DNA biosensor; (**a**) silica nanospheres (SiNSs) with gold nanoparticles (AuNPs); (**b**) SiNSs, AuNPs, glutaraldehyde; (**c**) after immobilization with DNA probe; (**d**) after hybridisation with complementary DNA; (**e**) after intercalation with HexaFc.

FESEM examined the morphology of the DNA biosensor. FESEM can provide a clear view of the structure of the DNA biosensor. Mapping with EDX made it possible to view the specifics of the DNA biosensor behaviour. Particularly after immobilisation of the aminated probes and hybridisation with complementary DNA. Figure 7 shows the morphology of the stepwise fabrication of the DNA biosensor. From the FESEM micrograph (Fig. 7a), SiNSs were spherical with diameter between 20 and 200 nm while AuNPs with diameter less than 100 nm. Both SiNSs and AuNPs were dispersed with high homogeneity. More DNA probes could be immobilised on silica nanospheres than flat surface silica, hence increase the biosensor sensitivity^32^. The DNA hybridisation in the presence of complementary DNA involving the intercalation with hexaferrocenium complex can be confirmed from FESEM studies (Fig. 7). Thus, a noticeable change on the biosensor surface before and after interaction with a complementary porcine DNA was observed.

The dispersive energy X-ray (EDX) elementary mapping analysis was employed to detect the distribution of the different elements present in the biosensor^33^. This biosensor consists of carbon, oxygen, silicon and aurum elements. The elemental mappings of Au (yellow), C (red), Si (blue) and O (green) were observed and have shown to be well distributed in the biosensor (Fig. 7a,b). As can be seen, nitrogen element was appeared after DNA immobilisation (Fig. 7c) and it is derived from the aminated DNA probe (N–H). The presence of the nitrogen atom on the biosensor caused a higher attenuation of X-ray and gave a better contrast on the image. The N element increases in Fig. 7d due to the formation of double-stranded DNA^34^. The process of DNA hybridisation is successful and was proved by the addition of a nitrogen element in Fig. 7d. Meanwhile, Fig. 7e shows the presence of Fe element after HexaFc complex intercalation is performed. The combination of FESEM micrograph and EDX mapping supports the observation of stepwise fabrication of DNA biosensor, including DNA immobilisation and DNA hybridisation process.

### Electrochemical studies

**Figure 8:**
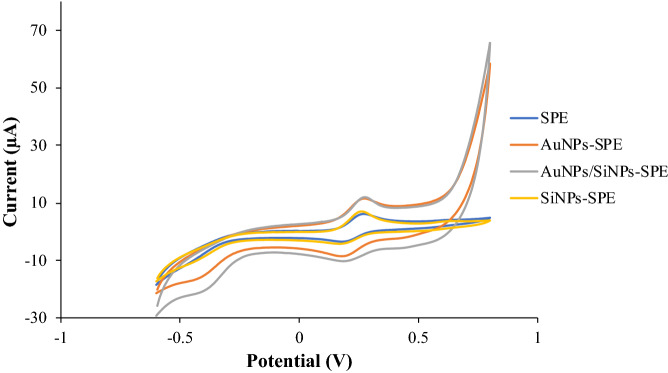
Cyclic voltammograms of 2 × 10^−4^ M HexaFc complex from bare SPE (SPE), gold nanoparticles modified SPE (AuNPs-SPE), gold nanoparticles/silica nanospheres modified SPE (AuNPs/SiNSs-SPE), and silica nanospheres modified SPE (SiNSs-SPE) at a scan rate of 80 mVs^−1^.

The electrochemical study of HexaFc complex solution was inspected by cyclic voltammetry (CV) to determine their electron transfer properties. Figure 8 shows every layer of modified SPE that were tested by CV with HexaFc complex solution as a redox-active test probe. The sigmoid curve from the biosensor response proposed that the majority of radial diffusion occur on the electrode surface^35^. Table 1 shows the peak separation between anodic and cathodic peak potential (ΔEP) and anodic and cathodic peak current ratio (IPA/IPC). The separation between anodic and cathodic peak relates to ion resistance involved in the redox reaction^36^.

**Table 1:** Electrodynamics data about anodic peak potential (E_PA_), cathodic peak potential (E_PC_), potential difference (∆E_p_), anodic peak current (I_PA_), cathodic peak current (I_PC_) and anodic to cathodic peak current ratio (I_PA_/I_PC_) of HexaFc with different surface-modified working electrodes.

	*E*_PA_ (V)	*E*_PC_ (V)	∆*E*_P_ (V)	*I*_PA_ (A)	*I*_PC_ (A)	|*I*_PA_/*I*_PC_|
Bare SPE	0.26566	0.18524	0.08042	4.5673 × 10^−6^	− 3.0541 × 10^−6^	1.4955
AuNPs- SPE	0.26566	0.18997	0.07569	6.4508 × 10^−6^	− 7.0527 × 10^−6^	0.9147
SiNSs-SPE	0.26093	0.17578	0.08515	5.9079 × 10^−6^	− 3.0966 × 10^−6^	1.9079
AuNPs/SiNSs-SPE	0.27039	0.19943	0.07096	6.1910 × 10^−6^	− 6.1335 × 10^−6^	1.0094

Table 1 display the potential difference (∆EP) increase in the electrode order of AuNPs/SiNSs-SPE < AuNPs-SPE < bare SPE < SiNSs-SPE as the electron transfer rate decreased at the electrode surface in the electrode order of AuNPs/SiNSs-SPE > AuNPs-SPE > bare SPE > SiNSs-SPE. As the electron transfer rate decreased, CV became more expansive, and ΔEp value increased. According to Monk^37^, the reversible redox system can be determined by peak potential differences and IPA/IPC values ~ 1. Anodic peak current (I_pa_) values increase in the electrode order of bare SPE < SiNS-SPE < AuNPs/SiNS-SPE < AuNPs-SPE, were recorded. A large increase in I_pa_ is due to the enhanced electro active surface are and electron transfer ability. Redox mediator facilitates the electron transfer process during the electrochemical reaction^38,39^.

The bare SPE and the SiNSs-SPE depicted greater peak separations. This may be attributed to the sluggish electron conductivity of the bare electrode, and the non-conductive properties of the SiNSs, which resulted in low electron transfer on the electrode surface. Therefore, the difference between anodic and cathodic peaks can be an indication of the resistance of electron transfer of the electrode^40^. The value of ΔEP generally decreases due to the presence of AuNPs of good electrical properties because AuNPs enhanced the electron transfer rate^41^. The current oxidation-reduction peak (IPA/IPC) ratio of AuNP/SiNSs-SPE was close to 1. It shows that this system is still reversible even though the electrode has been modified.

**Figure 9:**
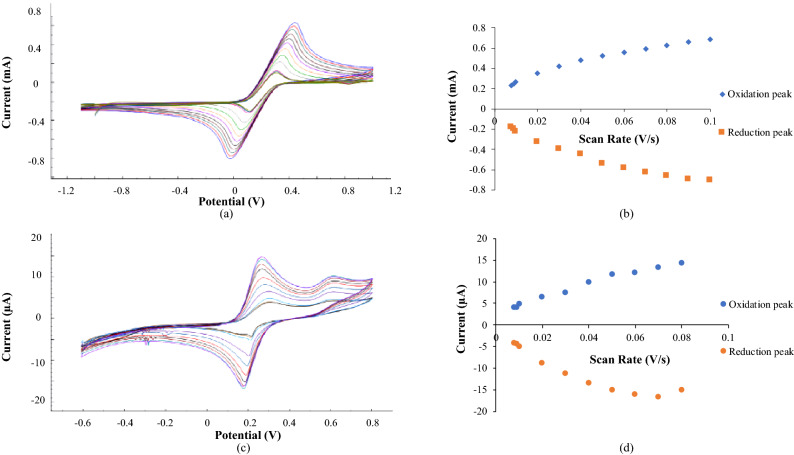
(**a**) scan rate study of DNA biosensor in K_3_Fe(CN)_6_; (**b**) cyclic voltammetry plot at different scan rate in K_3_Fe(CN)_6_; (**c**) scan rate study of DNA biosensor in HexaFc complex solution and (**d**) cyclic voltammetry plot at different scan rates in HexaFc complex solution.

The scan rate study was conducted from 0.008 until 0.1 V s^−1^ in the K_3_[Fe(CN)_6_] redox indicator (Fig. 9a). The K_3_[Fe(CN)_6_] system is an appropriate and valued tool for monitoring the characteristics of the modified electrode^37^. According to Fig. 9a, the peak current for oxidation and reduction increases proportionally with the scan rate from 0.008 until 0.1 V s^−1^. Figure 9b demonstrates the cyclic voltammetry plot at different scan rates. Polarisation increase with the increasing of scan rate, and it develops wide and distorted oxidation and reduction peak. Other than that, the increasing scan rate will lead to the increasing of voltage change among anodic and cathodic peaks^42^.

Scan rate study was also performed in HexaFc complex from 0.008 until 0.08 V s^−1^ to investigate the electrochemical process of the complex. Figure 9c displays that the redox peak current increases with the scan rate from 0.008 until 0.08 V s^−1^. However, there is no much difference in the redox peak current for the scan rates within 0.09 and 0.1 V s^−1^. This result suggests that HexaFc complex is irreversible at the higher scan rate. The scan rates from 0.008 until 0.08 V s^−1^ were more reversible, proof by the linear increase in the current redox (Fig. 9d). According to the larger peak area of the CV curves, more material was set based on the Faraday Law^43^.

### Analytical performance

SiNSs is a non-conductive material and has been used as DNA probe immobilisation sites. The low conductivity of SiNSs reduces biosensor performance. AuNPs have therefore been used to overcome this problem. AuNPs can enhance the potential of electron transfer from the redox indicator and improve the biosensor sensitivity^40^. Glutaraldehyde has been utilised as a link between porcine DNA probe and to the amine groups of SiNSs. The pH buffer also plays an essential role in producing DNA biosensor because it provides a suitable environment for DNA hybridisation process^32,43^. Generally, an acidic or basic environment can cause DNA damage^32^. All the parameters in the optimised condition were used to fabricate porcine DNA biosensor and shown in Table 2.

**Table 2:** Optimised parameter of porcine DNA biosensor.

Parameters	Optimum amount
Amount of gold nanoparticles	0.05 mg
Amount of silica nanospheres	0.04 mg
% of glutaraldehyde	10
Potassium phosphate buffer concentration	0.05 M
Potassium phosphate buffer pH	pH 7
Ionic strength	1 M
Concentration of DNA probe	1 µM
Immobilization time	24 h
Concentration of HexaFc complex	2 × 10^−5^ M

**Figure 10:**
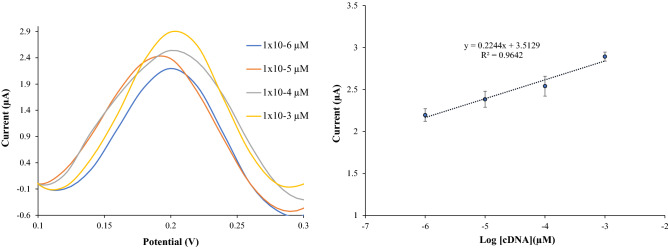
Linear response range for porcine DNA biosensor.

**Figure 11:**
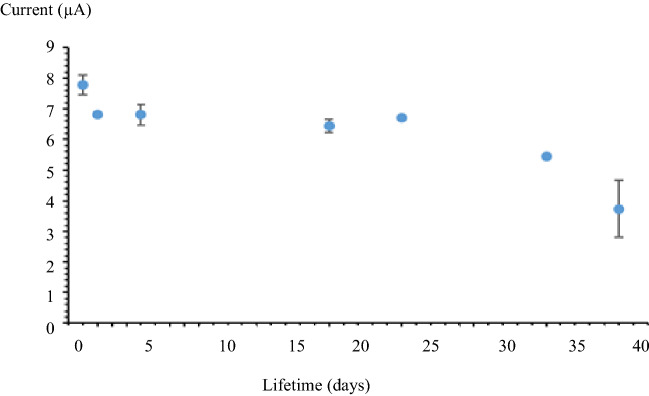
Shelf life of porcine DNA biosensor.

The performance of the biosensor was tested in a sodium phosphate buffer solution, 0.05 M containing complementary porcine DNA with different concentration and 1 M Na^+^ ion at pH 7. Figure 10 shows a good linearity response of complementary porcine DNA from 1 × 10^−6^ µM until 1 × 10^−3^ µM with the correlation coefficient, R^2^ = 0.9642. Limit of detection (LOD) for complementary porcine DNA was determined at 4.83 × 10^−8^ µM. The LOD of the porcine DNA biosensor was calculated following the three times of the standard deviation of the biosensor response in the linear range divided by the direct calibration code^44^. The average reproducibility relative standard deviation for each calibration point of this biosensor is excellent with most values below 4.5% (n = 3). The shelf life of the porcine DNA biosensor is shown in Fig. 11. The fabricated biosensor was store at 4 °C before tested. From the study, the DNA biosensor response did not show any difference from day 1 until day 23. The percentage response is between 90 and 86% compare to day 1. Day 33 until day 38, the biosensor response decreases until 70–50% due to the degradation either from probe DNA, AuNP or SiNS from the electrode. The degradation effects reduce the quantity of immobilized probe DNA and reduce the biosensor response.

**Figure 12:**
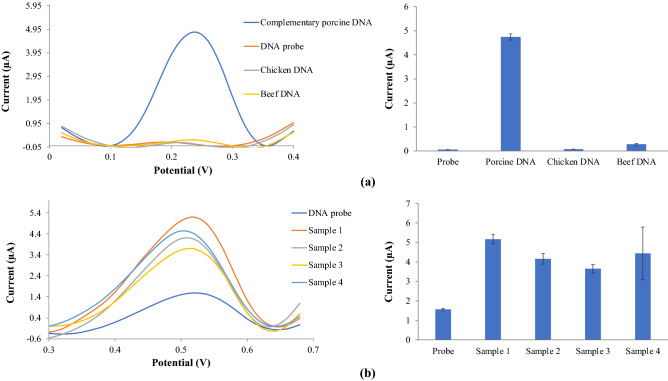
(**a**) porcine DNA biosensor selectivity on complementary porcine DNA, chicken DNA and beef DNA and (**b**) real samples measured with porcine DNA biosensor.

Figure 12a exhibits the DPV response before and after the probe-complementary DNA hybridisation process. The DNA electrode based on AuNPs/SiNSs-SPE showed a 100% matching of the complementary-porcine DNA sequences. The strong and sharp of the DPV peak at a potential ~ 0.22 V revealed that the HexaFc complex had been successfully intercalated into the porcine DNA double-stranded and it suggested that DNA probe is immobilised and hybridised with complementary-DNA. DNA probe covalently immobilised with SiNSs. No DPV peak was found at potential ~ 0.22 V for single-stranded porcine DNA probe, and only a small peak was obtained after this biosensor exposed to beef and chicken DNA. This new research will be beneficial for determining porcine DNA in food products by applying new HexaFc complex as an electrochemical porcine DNA indicator.

This DNA biosensor was tested with raw pork meat from several markets and supermarkets around Bangi, Malaysia. It was discovered that the signal from raw pork was found to have a high current value compared to a DNA probe (Fig. 12b).

## Performance comparison with other reported porcine DNA biosensors

Table 3 displays a performance comparison between the constructed porcine DNA biosensor and the other electrochemical porcine DNA biosensors. This comparison is needed for the validation of the constructed biosensor. The constructed porcine DNA biosensor showed substantial improvements in high linear range response and low detection limit compared to other DNA immobilisation matrixes, such as disposable electrochemical printed chips^45^, gold nanoparticles^46^, AuNP/NBA-NAS^47^ and graphene biochips^48^. This outcome is attributed to the large surface area of silica nanospheres (SiNSs) for DNA immobilisation sites. When compared with DNA hybridisation indicator that employed ferrocenium compounds^9,10^, the biosensor developed here has comparable LOD, but it did not require any procedure for ferrocene labelling, i.e. chemical attachment of ferrocene compounds to the DNA. This advantage is because the ferrocenium complex used in this work detects DNA hybridisation by intercalation.

**Table 3:** Comparison of performance between the DNA biosensor reported here with other biosensors using different ferrocene and non-ferrocene based compounds as an indication of hybridisation.

DNA electrode materials	DNA label	Linear range (µM)	LOD (µM)	References
SPE/AuNPs/SiNSs	HexaFc complex	1 × 10^−6^–1 × 10^−3^	4.83 × 10^−8^	This work
Fc-acid-OMPA	Ferrocene oligomer	1.0 × 10^−9^–1.0 × 10^−4^	2.0 × 10^−9^	^9^
Graphene	Tetraferrocene	2.0 × 10^−8^–2.0 × 10^−3^	8.2 × 10^−9^	^10^
Gold nanoparticles-DNA bioconjugate	Methylene blue	7.35 × 10^−3^–3.68 × 10^−1^	4.26 × 10^−2^	^43^
SPE/AuNP/NBA-NAS	Ruthenium(II) complex	1.0 × 10^−7^–1.0 × 10^−2^	**–**	^44^
Graphene biochips	Ruthenium hexamine	–	6.89	^45^
Disposable electrochemical printed chips	Hoechst 33258	–	1.34	^42^

## Conclusion

The new HexaFc complex has demonstrated good DNA binding and selectivity from spectrophotometric studies. It was then successfully used as a new redox indicator for an electrochemical DNA biosensor to determine porcine DNA. Most importantly, the DNA biosensor with this ferrocenium indicator showed a good response towards complementary porcine DNA. It is potentially an easy and rapid method for the determination of porcine DNA in food products.
